# Pharmacokinetics of single dose radium-223 dichloride (BAY 88-8223) in Japanese patients with castration-resistant prostate cancer and bone metastases

**DOI:** 10.1007/s12149-016-1093-8

**Published:** 2016-06-07

**Authors:** Keisuke Yoshida, Tomohiro Kaneta, Shoko Takano, Madoka Sugiura, Tsuyoshi Kawano, Ayako Hino, Tou Yamamoto, Kazuya Shizukuishi, Masato Kaneko, Christian Zurth, Tomio Inoue

**Affiliations:** 1 Department of Radiology, Yokohama City University, 3-9 Fukuura, Yokohama, 236-0004 Japan; 2 Department of Radiation Oncology/Diagnostic Radiology, Tohoku University Hospital, Sendai, Japan; 3 Radiation Oncology, Shonan Kamakura General Hospital, Kamakura, Japan; 4 Department of Diagnostic Radiology, Yokohama City University Medical Center, Yokohama, Japan; 5 Saitama Central Clinic, Saitama, Japan; 6 Clinical Sciences Japan, Bayer Yakuhin Ltd., Osaka, Japan; 7 Clinical Sciences, Bayer Pharma AG, Berlin, Germany

**Keywords:** Radium-223, Castration-resistant prostate cancer, Metastases, Pharmacokinetics

## Abstract

**Objective:**

This open-label, non-randomized, phase I study examined the pharmacokinetics (PK) and radiation dosimetry of a single dose of radium-223 in Japanese patients with castration-resistant prostate cancer (CRPC) and bone metastases.

**Methods:**

Six male Japanese patients (mean age 72.5 years, range 65–79 years) with histologically or cytologically confirmed stage IV adenocarcinoma of the prostate were recruited. A single IV dose of radium-223 was delivered intravenously (IV) via slow bolus over a 2–5 min period: Cohort 1 received 50 kBq/kg and Cohort 2 received 100 kBq/kg.

**Results:**

Following IV injection, radium-223 was rapidly eliminated from the blood in a multi-phasic manner. The fraction of the injected activity of radium-223 retained in the whole body 24 h following injection was 85 %. Biodistribution results showed initial bone uptake was 52 % (range 41–57 %). The maximum activity of radium-223 in the bone was observed within 2 h of dosing. Activity of radium-223 passed through the small intestine within 24 h. No activity was detected in other organs. The major radiation dose from radium-223 was found in osteogenic cells; calculated absorbed doses in osteogenic cells and in the red marrow were 0.76 Gy/MBq and 0.09 Gy/MBq, respectively.

**Conclusions:**

In Japanese patients with CRPC and bone metastases, radium-223 (IV) achieved maximum activity in the bone rapidly and passed through the intestine within 24 h, without signs of activity in other organs. The PK profile and absorbed radiation dose in organs and tissues in Japanese patients were similar to data from non-Japanese patients.

Trial registration identification: NCT01565746.

## Introduction

There are limited treatment options for men with castration-resistant prostate cancer (CRPC). The prognosis is particularly poor in those with bone metastases, and until recently, therapy consisted of cytotoxic chemotherapy or palliative therapy to maintain quality of life [1].

Radium-223 is a first-in-class radioactive alpha particle emitting agent and the first radiopharmaceutical to demonstrate an improvement in overall survival and reduced time to first symptomatic skeletal-related event in patients with CRPC with symptomatic bone metastases [2–5]. Radium-223 is calcium mimetic, and therefore, targets locations with high calcium turnover, such as bone, thereby delivering cytotoxic radiation directly to bone metastases [6]. Due to the targeted mechanism of action, radium-223 has an improved toxicity and safety profile compared with non-targeted agents [7].

Aside from radium-223, strontium-89 is also an alkaline earth metal that mimics calcium and is a known radiopharmaceutical for the management of various cancers. Strontium-89 is a beta emitter while radium-223 is an alpha emitter. Compared with strontium-89, radium-223 has a shorter physical half-life (11.4 versus 50.5 days), shorter tissue range (<0.1 versus 8 mm) and higher linear energy transfer, meaning that radium-223 is more likely to target tumor cells while minimizing toxicity to healthy bone marrow [8–10]. In an in vivo murine study, both strontium-89 and radium-223 were concentrated on bone surfaces but bone uptake of radium-223 was slightly higher than that of strontium-89. Radium-223 had little redistribution of daughter products from bone, suggesting it remains at the target site and delivers intense and focused radiation doses to bone surfaces, delivering less radiation to non-target tissue than strontium-89 [8].

Intravenous (IV) radium-223 was approved as first-line therapy in patients with CRPC symptomatic bone metastases and no known visceral metastatic disease, by the US Food and Drug Administration in May 2013 and by the European Medicines Agency in November 2013 [11]. It is currently in phase III clinical development in Japan for the treatment of bone metastases (https://www.clinicaltrialsregister.eu/ctr-search/trial/2013-003438-33/SE). Pharmacokinetic (PK) studies of radium-223 in non-Japanese patients have shown that it is rapidly cleared from the blood, taken up by bone with very little organ distribution and eliminated mainly via fecal elimination [12, 13]. In the blood, the activity level of radium-223 decreases to ~1 % of the initial value after 24 h [14]. However, little is known regarding the PK properties of radium-223 in Asian patients. Therefore, the present study was performed to describe the PK and radiation dosimetry of a single dose of radium-223 in Japanese patients with CRPC and bone metastases.

## Materials and methods

### Study design

This open-label, non-randomized, phase I study assessed the PK, biodistribution and radiation dosimetry of a single IV dose of radium-223 when delivered via slow bolus (Trial registration identification: NCT01565746). This was a single center study; eligible patients were recruited from Yokohama City University Hospital in Japan. This study was approved by the Yokohama City University Hospital Institutional Review Board (Chairperson Tomoyuki Saito) and all participants provided signed informed consent.

### Study participants

The study recruited male patients aged ≥20 years with histologically or cytologically confirmed adenocarcinoma of the prostate. Patients were required to have more than two scintigraphically confirmed bone metastases within the previous 4 weeks and have previously failed initial hormone therapy. Eligible patients had progressive disease as evidenced by at least one of the following: new osseous lesions, ≥20 % increase in the sum of the longest diameter of target lesions or biochemical progression (≥3 rising prostate serum antigen values from baseline obtained ≥1 week apart or 2 measurements ≥2 weeks apart or 2 measurements ≥2 weeks apart). Other inclusion criteria were: life expectancy of ≥8 weeks, Eastern Cooperative Oncology Group performance status 0–2, able to tolerate scanning for ≥30 min, surgical sterilization or using adequate contraceptive precautions during treatment and for 3 months after the last administration of radium-223.

Patients were excluded if they had received chemo-, immuno- or radiotherapy within 4 weeks prior to study entry, required immediate external beam radiotherapy, had a history of gastrointestinal (GI) bleeding or ulcer within the past 3 months, required oxygen for pulmonary metastases, had other currently active malignancies, apart from prostate cancer-related metastases or had a bone fracture in the healing stage.

### Drug treatment

Patients were assigned, in a non-randomized design, to either a single dose of 50 kBq/kg [55 kBq/kg after implementation of NIST update [15]; Cohort 1 (*n* = 3)] or a single dose of 100 kBq/kg (110 kBq after implementation of NIST update [15]) of radium-223 [Cohort 2 (*n* = 3)] as an IV infusion over a period of 2–5 min. The 100 kBq/kg dose was included in the study for the purpose of obtaining good biodistribution data (since higher doses are associated with better quality images).

### Study assessments

Whole body radioactivity was measured using a 2 m accurate radioisotope counting detector and gamma scintigraphy. The total activity concentrations in blood and plasma were expressed in kBq/mL, and subsequently, normalized by the concentration at the time of injection extrapolated from the first two data points to obtain the fraction of injected activity (%ID) at each sampling time. PK parameters were calculated model independently using the software WinNonlin. Sampling for biodistribution assessment after the first administration was performed for up to 72 h for blood and feces, 48 h for urine, and for up to 8 days for whole body and multiple organ assessments. Sampling for assessment of blood kinetics (approximately 4 mL blood) was performed at different time points after study drug administration, from a vein in the contralateral arm to the injection site. Whole blood and plasma samples were counted collectively after the completion of all sampling. Urine was collected pre-dose, 0–4, 4–8, 8–24 and 24–48 h post-dose. The radioactivity in whole blood, plasma and urine was determined in a calibrated NaI well-type detector alongside the radium-223 reference standard made up to the same volume (1 mL) as the blood, plasma and urine samples. The number of counts was corrected for background and converted into activity via a measured calibration factor. These values (kBq/mL) were further corrected back for decay until the time of administration. The cumulative amount of radium-223 excreted with urine and feces was determined as a proportion of the injected dose.

Biodistribution (image processing and activity quantification) and radiation dosimetry were conducted by an experienced nuclear medicine expert and a dosimetry expert, respectively. All images were taken using the Symbia T-16 gamma camera and all gamma camera image processing was carried out on the Hermes system supplied by Hermes Medical Solutions; the energy window for the images was 81 keV. The count rate for all measurements was sufficiently low that no correction was required for detector dead time. Activity quantification was performed on patient images according to the conjugate view method. The mirrored posterior-anterior image was multiplied with the anterior-posterior image, and the geometric mean was calculated. Regions of interests (ROIs) were drawn and the activity measured was corrected for activity in over- and underlying tissues. The size of each region and the number of counts in the region were recorded. Area normalized background subtraction was carried out on the counts from each ROI, and the geometric mean of the corrected anterior and posterior counts was then determined. The cumulative activity (CA) (MBq × h) in the source organs was derived from the whole body subject scans for all organs with quantifiable amounts of activity in the images. The CA for a particular organ was the integral of the time activity curve for that organ obtained, from imaging or tissue sampling.

For dosimetry, absorbed doses were estimated using the OLINDA EXM^®^, software based on the Medical Internal Radiation Dose algorithm, which is widely used for established beta and gamma emitting radionuclides. The absorbed doses were calculated for radium-223 and all its daughter nuclides assuming that their decay took place at the site of parent decay. For radium-223, which is primarily an alpha-emitting radionuclide, additional assumptions were made for the intestine, red marrow and bone/osteogenic cells, to provide the best possible absorbed dose calculations for radium-223 dichloride, considering its observed biodistribution and specific characteristics of radium-223 dichloride as an alpha particle emitter.

Those patients with large metastases or a large number of metastases (i.e., superscan) were excluded from dosimetric assessment due to an artificial impact their disease state would have on the determination of whole body activity.

### Statistical analysis

All patients who received at least one dose of study medication and had valid data for the PK analysis were included in the PK evaluation. Demographic and other baseline characteristics were summarized using descriptive statistics, such as frequency and proportion (for categorical variables), mean, standard deviation, median and minimum/maximum (for continuous variables).

The time activity curves in blood and plasma were summarized separately for each treatment group. The total radioactivity in urine (kBq/mL), urinary and fecal excretion amount of total radioactivity (kBq) and excreted radioactive dose (%ID) were summarized separately for each treatment group. Statistical analyses were performed for each collection period.

The biodistribution and dosimetry data were listed by subject and presented using descriptive statistics.

## Results

### Study participants

Six Japanese patients were enrolled (mean age 72.5 years, range 65–79 years) and evenly distributed between Cohorts 1 and 2. All patients had stage IV CRPC and median Gleason score 9.00; median time since diagnosis was 48 months. Baseline patient characteristics are shown in Table 1.

**Table 1:** Baseline demographic characteristics of the study participants

	(Cohort 1, *N* = 3)			(Cohort 2, *N* = 3)		
	Patient 1	Patient 2	Patient 3	Patient 4	Patient 5	Patient 6
Age (years)	66	75	79	76	65	74
Weight (kg)	72.5	65.6	65.0	56.5	61.5	62.2
Extent of disease (EOD grade)	2	2	1	1	3	2
Dosing arm (kBq/kg)^a^	50	50	50	100	100	100
Injected activity (MBq)^b^	4.01	3.54	3.43	5.78	5.74	6.52
Injected activity (kBq/kg)^b^	55.31	53.92	52.77	102.37	93.33	104.79

*EOD* extent of disease

^a^The dosing arm 50 and 100 kBq/kg are 55 and 110 kBq/kg after implementation of NIST update [12], respectively

^b^The corresponding values after implementation of NIST update [12] can be obtained by multiplying the current values by 1.105

### Drug presence and elimination from the blood

**Fig. 1:**
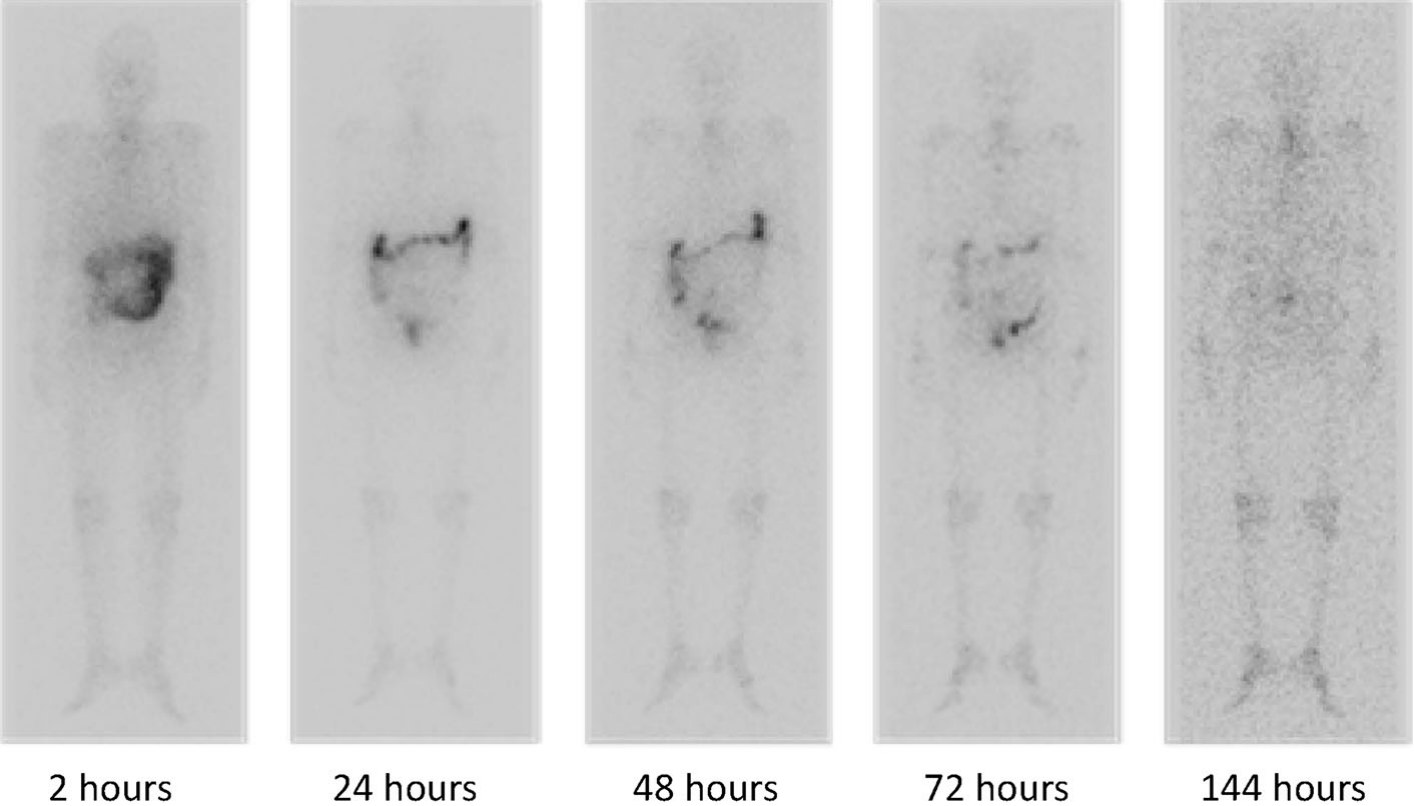
Biodistribution of activity, as detected via gamma camera, of radium-223 over time in patient #4

**Fig. 2:**
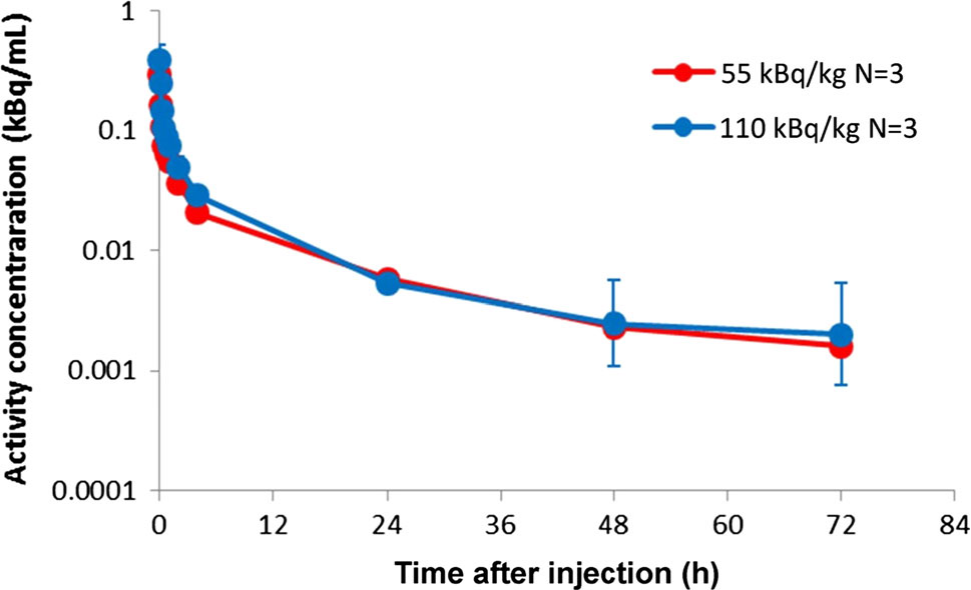
Pharmacokinetic profiles of activity concentration in blood after single injection of radium-223. The dosing arm 50 and 100 kBq/kg are 55 and 110 kBq/kg after implementation of NIST update [12], respectively

Approximate dose proportionality was observed between the two doses: maximum serum concentration/dose [geometric mean: Cohort 1: 0.080 (1/L), Cohort 2: 0.064 (1/L)] and area under the concentration versus time curve/dose were comparable between the two cohorts (geometric mean: Cohort 1: 0.157 h/L, Cohort 2: 0.115 h/L). Figure 1 illustrates the biodistribution/gamma camera images of radium-223 over time. Following IV injection, radium-223 was rapidly eliminated from the blood in a multi-phasic manner (Fig. 2). The fraction of the injected activity retained in the blood at 15 min following injection was 22 % (range 9–28 %). At 4 h after the injection, only 4 % (1–6 %) of the injected activity remained in the blood, which decreased to 0.3 % (0.0–0.9 %) 72 h after the injection.

### Excretion

**Fig. 3:**
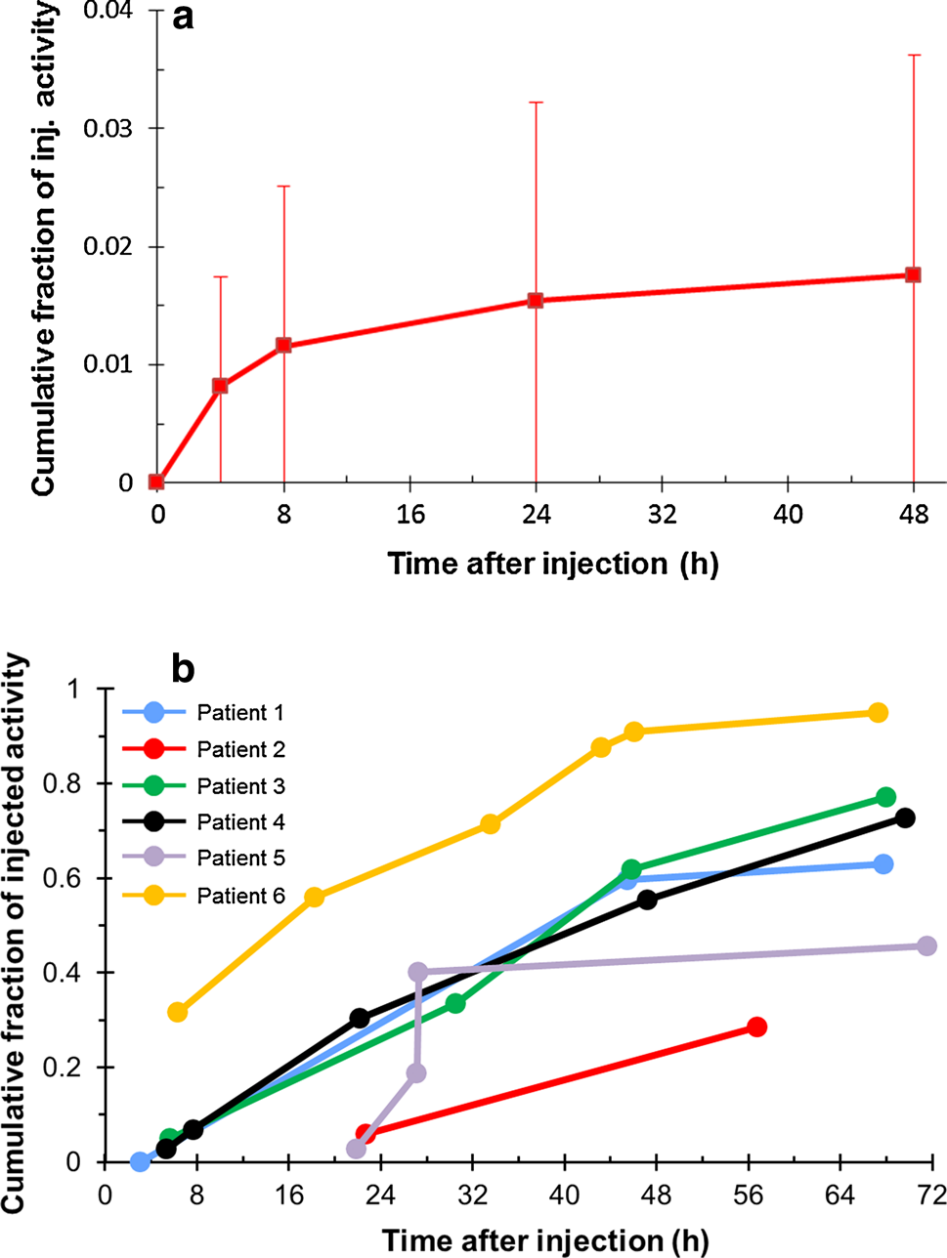
Arithmetic mean percentage (standard deviation) of the cumulative activity excreted after injection of radium-223 in the **a** urine and **b** feces as a proportion of the injected dose. Fraction of injected activity represents the proportion of radioactivity (as a proportion of the injected dose) that was detected at various time points. Patients 1–3 received 50 kBq/kg (55 kBq/kg after the NIST update [12]) and patients 4–6 received 100 kBq/kg (110 kBq/kg after the NIST update [12])

Urine excretion was minimal; cumulative urine excretion up to 48 h after administration reached 2 % (Fig. 3a), while cumulative fecal excretion reached 64 % (29–95 %) up to 72 h after administration (Fig. 3b).

### Biodistribution

#### Whole body

**Fig. 4:**
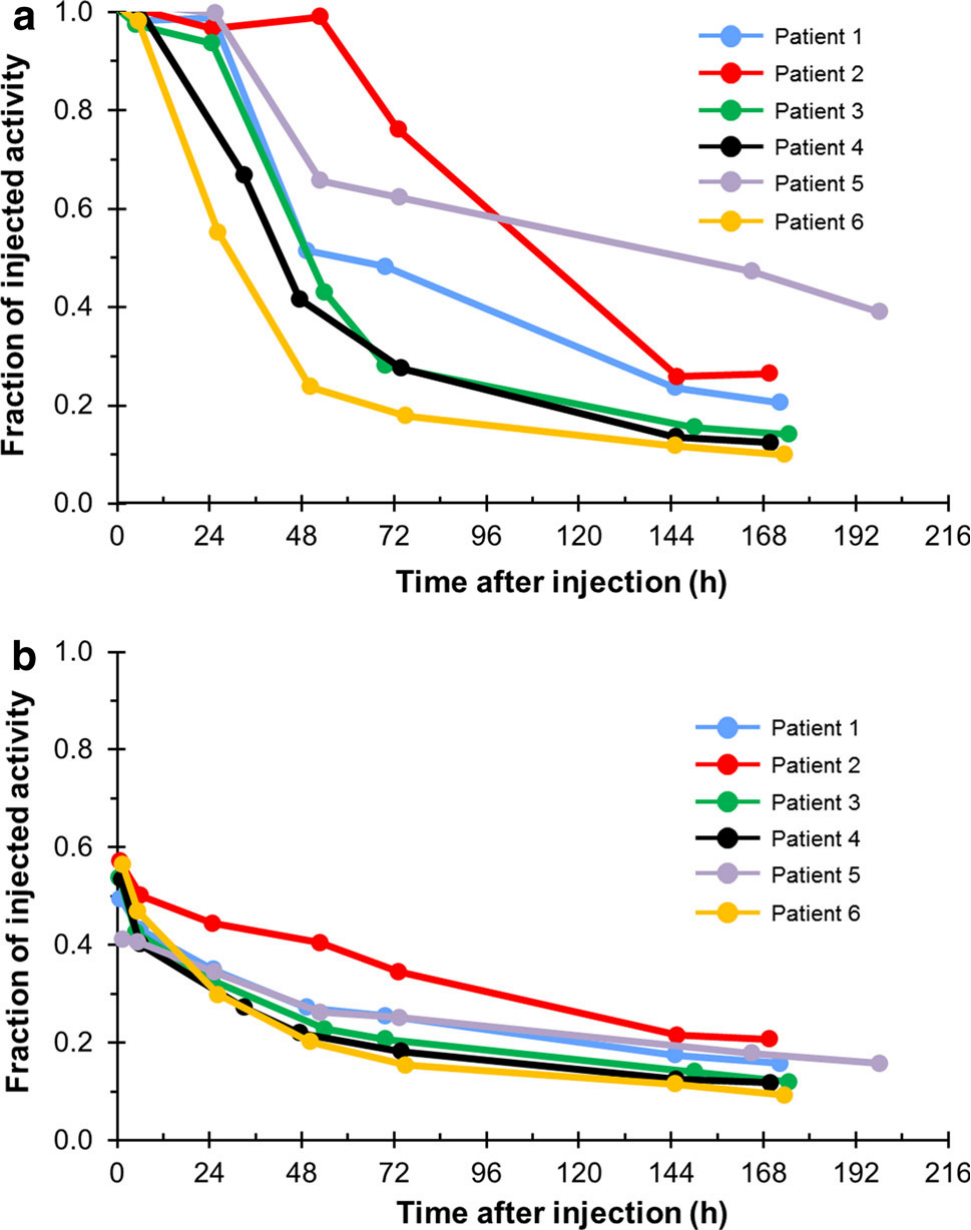
Fraction of injected activity in **a** the whole body and **b** the bone after injection of radium-223. Fraction of injected activity represents the proportion of radioactivity (as a proportion of the injected dose) that was detected at various time points. Patients 1–3 received 50 kBq/kg (55 kBq/kg after the NIST update [12]) and patients 4–6 received 100 kBq/kg (110 kBq/kg after the NIST update [12])

The fraction of the injected activity of radium-223 retained in the whole body 24 h following a single injection was 85 % (range 55–100 %); this had decreased to 22 % at day 8 (Fig. 4a).

#### Bone

Biodistribution results showed rapid uptake of radium-223 in bone: initial bone uptake within 2 h was 52 % (range 41–57 %). The maximum activity of radium-223 in the bone was observed within 2 h of dosing (Fig. 4b).

#### Gastrointestinal (GI) tract

**Fig. 5:**
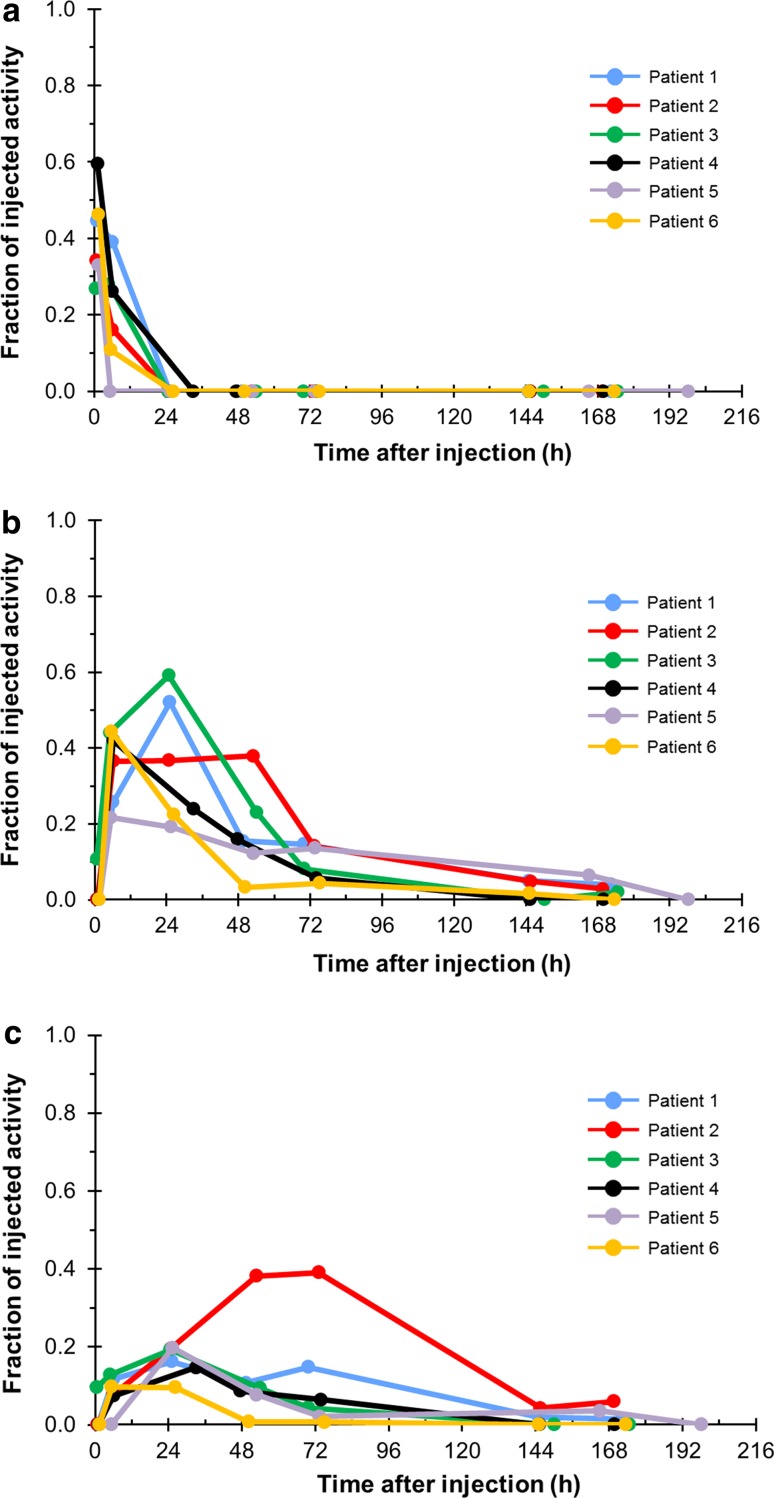
Fraction of drug activity in the **a** small, **b** upper large and **c** lower large intestines over time after injection of radium-223. Fraction of injected activity represents the proportion of radioactivity (as a proportion of the injected dose) that was detected at various time points. Patients 1–3 received 50 kBq/kg (55 kBq/kg after the NIST update [12]) and patients 4–6 received 100 kBq/kg (110 kBq/kg after the NIST update [12])

Activity of radium-223 passed through the small intestine (SI) within 24 h (Fig. 5). Six hours after injection, 64 % (range 22–85 %) of the activity was seen in the GI tract including the SI, upper large intestine (ULI) and lower large intestine (LLI). This decreased to 52 % (range 32–78 %) at 24 h, 31 % (4–76 %) at 48 h and 21 % (5–53 %) at 72 h. Eight days after the injection, radium-223 activity in the GI tract was 3 % (range 0–9 %). No specific activity was detected in other organs. No measurable activity was observed in the kidney after the first few hours post-injection.

#### Radiation dosimetry

Patient 2 and patient 5 were regarded as outliers due to the impact of large metastases or a high number of metastases on the calculation of individual organ doses.

The mean absorbed doses for different organs, excluding these two patients, are presented in Table 2.

**Table 2:** Mean absorbed doses (*N* = 4)

Organ/tissue	Mean (mGy/MBq)	%CV
Osteogenic cells	761	17
Red Marrow	91.6	17
Upper large intestine	24.4	41
Lower large intestine	18.8	44
Whole body	14.0	20
Small intestine	5.42	34
Kidneys	2.00	17
Liver	1.87	17
Urinary bladder	1.54	84
Heart	0.954	34
Ovaries	0.269	36
Gallbladder	0.151	35
Uterus	0.144	33
Stomach	0.0775	32
Adrenals	0.0635	20
Muscle	0.0605	25
Pancreas	0.0604	26
Brain	0.0498	17
Spleen	0.0439	26
Testes	0.0330	27
Lungs	0.0323	18
Thyroid	0.0318	17
Skin	0.0313	21
Thymus	0.0223	17
Breasts	0.0169	18

The majority of the radiation dose from radium-223 was found in osteogenic cells on bone surfaces. The calculated absorbed doses in the bone (osteogenic cells) and in the red marrow were 0.76 Gy/MBq and 0.09 Gy/MBq, respectively.

While the most dominant excretion organ was the GI tract, absorption of radium-223 in this organ was low (Fig. 5). Levels of absorbed doses in the SI, ULI and LLI walls were 0.005, 0.024 and 0.019 Gy/MBq, respectively.

## Discussion

This open-label study indicates that in Japanese patients with CRPC and bone metastases, radium-223 activity passed rapidly from blood into bone or into the small intestine, with no activity detected in other organs.

The patients enrolled in this study had poor prognostic factors at baseline: 84.2 % of patients had ≥6 metastatic sites, and 100 % had received prior systemic anti-cancer chemotherapy for adjuvant or palliative use. Following a single IV dose of radium-223, dose proportionality was roughly observed across both cohorts—although the number of patients in each cohort was limited low.

The results observed in Japanese patients are comparable with previously published data in Caucasian populations (study ATI-BC [12], study BC1-05 [14] and study BC1-08 [13]) with regard to the PK profile, urine excretion, whole body measurements, initial bone uptake and radioactivity in the GI tract. These three studies showed that in Caucasian patients, single dose IV radium-223 was rapidly cleared from the blood and <1 % remained after 24 h [12–14]; in the present study, the fraction of the injected activity retained in blood declined to <5 % after 4 h and <0.5 % after 72 h. The cumulative urine excretion was minor (2–4 %) in studies BC1-05 and BC1-08 [13, 14], while in the Japanese cohort, cumulative urine excretion up to 48 h after administration reached approximately 1 %. Whole body measurements at 7 days post-injection in study BC1-08 indicated that in Caucasian patients a median of 76 % of administered activity was excreted from the body (i.e., decreased to 24 % at 7 days) [13], while in Japanese patients whole body measurements decreased to 22 % at day 8. Scintigraphy demonstrated skeletal accumulation of radioactivity in Caucasian patients, with a preference for the osteoblastic metastases [12–14] which was also observed in Japanese patients in the present study. Biodistribution studies showed rapid transit of radium-223 into the SI, and in study BC1-05, 4 h post-injection the percentage of the radioactive dose present in bone was approximately 61 % [14]. After 24 h, a median of 52 % of the administered dose was in the LI in study BC1-08 [13]. Similarly, in the present study biodistribution results showed that activity was rapidly distributed into the bone, where the initial level of uptake was greater than 50 % at <2 h after injection; the maximum uptake of activity in the ULI was seen at or before 24 h, while the maximum uptake in the LLI occurred 24–72 h after injection.

In the absence of an established method to calculate the absorbed dose for alpha particle emitting radionuclides, different techniques give rise to slightly different results. The studies by Chittenden 2015 (study BC1-05) and Lassmann 2013 used different methods to calculate dosimetry data than were employed in our study [14, 16]. Nonetheless, there were no remarkable differences in the dosimetry findings between Japanese and non-Japanese patients based on the prescribing information and compared with previously published data [14, 16, 17]. In Japanese patients, the calculated absorbed doses in the bone (osteogenic cells) and in the red marrow were 0.76 and 0.09 Gy/MBq, respectively, compared with 0.76 and 0.08 Gy/MBq in non-Japanese patients, respectively [16] or 5.40 and <0.003 Gy/MBq, respectively [14] or 1.15 and 0.14 Gy/MBq, respectively [17]. The calculated absorbed doses in the GI tract (the dominant excretory organ) in Japanese patients were low: SIW, 0.0054 Gy/MBq; ULIW 0.0244 Gy/MBq; and LLIW, 0.0188 Gy/MBq. In non-Japanese patients, these values varied between 0.0036–0.0073, 0.0208–0.0380, and 0.0465–0.0610 Gy/MBq, respectively [14, 16, 17]. The results from this study, the published data and the prescribing information show that bone endosteum and red bone marrow have higher dose absorption coefficients than the GI tract. There is generally good agreement of the data in Japanese patients from this study with the data in non-Japanese patients from Lassmann et al. and the prescribing information [16, 17]. The difference seen for bone and red marrow in the publication from Chittenden et al. is probably due to different methods used for the dosimetry calculation for alpha particle emitting radionuclides [14].

The dosimetry results in Japanese and Caucasian subjects indicate that the estimated absorbed doses in both red marrow and osteogenic cells presented in this report are high; however, the safety data from phase I and II clinical trials in Japanese patients with radium-223 chloride are not consistent with such high absorbed doses to these organs, and the adverse event profile of Caucasian patients treated with radium-223 was similarly unremarkable [12–14]. This indicates that the estimated absorbed doses to these organs are very likely overestimated due to simplified assumptions regarding the cellular geometry relative to the range and spatial distribution of the alpha particle emissions [15]. The methodology to estimate dosimetry for alpha emitters is still at a very early stage and the available software developed for beta and gamma emitters does not apply.

Differences have been reported in the cumulative fecal excretion of radium-223 between Japanese (64 % up to 72 h after administration) and non-Japanese patients (13 % up to 48 h) [14], which are considered to be the result of the high variability in intestinal transit rates and frequency of bowel evacuation between Japanese and non-Japanese patients. However, there was no major difference in the elimination profiles from blood and plasma between Japanese and non-Japanese populations. Based on the biodistribution comparison, most Japanese and non-Japanese patients showed similar whole body radioactivity retention, within a range of 10–40 % at 7 to 8 days post-injection. In Japanese and non-Japanese populations, urinary excretion had made a small contribution to overall excretion of radioactivity, which was of no clinically relevant significance. Although differences in cumulative fecal excretion have been observed, this may be attributed to the difference in the residence time of intestinal contents in the GI tract and subsequent frequency of defecation. All radioactivity secreted in the GI tract is assumed to be excreted via the fecal route. Therefore, based on radioactivity detected at 24 h in the GI tract, it is considered that the definitive fecal excretion of radioactivity is generally similar in Japanese and non-Japanese patients. In addition, there are no significant differences in biodistribution and or dosimetry of radioactivity for organs/tissues between Japanese and non-Japanese populations.

It should be noted that the comparisons with the non-Japanese data (BC1-05) set are confounded by the fact that the non-Japanese study population included one patient of Asian ethnicity (within the USA). Prescribing Information and the Chittenden et al. 2015 article, the PK data from BC1-05 were obtained from six patients (five Caucasian and one Asian) after the first and second administration of 100 kBq/kg [14, 17]. Therefore, the comparisons are not made between strictly defined ethnicity groups.

While the results of the present study are somewhat limited by the small study population, these findings are strengthened by the use of robust analytical techniques and the long assessment period (up to 8 days) for PK analysis.

The results of the present study add to the existing literature and indicate that the PK profile and absorbed radiation dose in organs and tissues in Japanese patients with CRPC and bone metastases receiving a single dose of radium-223 are similar to data from non-Japanese patients.
